# Sedentary behavior, physical activity, psychological well-being, and gynecologic and obstetric disorders: A multivariable Mendelian randomization and mediation analysis

**DOI:** 10.1097/MD.0000000000048611

**Published:** 2026-05-08

**Authors:** Yide Hu, Yunbo Cao, Hong Wu, Bo Wang, Junxu Li, Weiting Xia, Bingdong Zhan

**Affiliations:** a School of Public Health, Zhejiang Chinese Medical University, Hangzhou, China; b Quzhou Center for Disease Control and Prevention, Quzhou, Zhejiang, China; c Hepatobiliary and Pancreatic Interventional Treatment Center, Division of Hepatobiliary and Pancreatic Surgery, The First Affiliated Hospital, Zhejiang University School of Medicine, Hangzhou, Zhejiang, China; d Institute of Translational Medicine, Zhejiang University School of Medicine, Hangzhou, Zhejiang, China; e Department of HIV/AIDS Control and Prevention, Hangzhou Center for Disease Control and Prevention (Hangzhou Health Supervision Institution), Hangzhou, Zhejiang, China; f The First Affiliated Hospital of Changchun University of Chinese Medicine, Changchun, Jilin, China; g Department of Gynecology and Obstetrics, The First Affiliated Hospital, Zhejiang University School of Medicine, Hangzhou, Zhejiang, China; h Department of Gynecology, The First Affiliated Hospital of Wenzhou Medical University, Wenzhou, Zhejiang, China.

**Keywords:** gynecologic and obstetric disorders, Mendelian randomization, movement behaviors, psychological well-being

## Abstract

The independent causal effects of interrelated lifestyle behaviors and psychological states on women’s gynecologic and obstetric health remain incompletely understood. We conducted a comprehensive Mendelian randomization (MR) study to disentangle the effects of leisure screen time, moderate-to-vigorous physical activity (MVPA), experiencing mood swings, and the well-being spectrum on a wide range of these disorders. We performed 2-sample MR analyses using data from the UK Biobank and FinnGen cohorts for 20 outcomes, with causal estimates combined via meta-analysis. The analytical framework included univariable MR, multivariable Mendelian randomization (MVMR) to assess independent effects, and an exploratory mediation analysis. A comprehensive suite of sensitivity analyses was used to assess the robustness of the findings against pleiotropy and heterogeneity, and the results were corrected for multiple testing using the false discovery rate. Our analyses identified a core set of 6 disorders, including polycystic ovaries, endometriosis, and menstrual disorders, that were robustly associated with all 4 exposures. Overall, genetically predicted leisure screen time and experiencing mood swings were causally associated with an increased risk for 10 and 12 disorders. Conversely, MVPA and well-being spectrum were associated with a decreased risk for 9 and 12 disorders. These associations were largely independent in MVMR analyses. Exploratory mediation analysis highlighted that inflammation (via C-reactive protein) and hormonal pathways (via estradiol) mediated numerous associations, such as the C-reactive protein-mediated protective effect of MVPA on female infertility. This study provides genetically strengthened evidence for independent causal relationships between movement behaviors, psychological states, and a wide spectrum of gynecologic and obstetric disorders. Methodologically, our MVMR approach was instrumental in disentangling the effects of correlated exposures. Mechanistically, our findings point toward inflammation and hormonal pathways as potential mediators, which may inform future prevention and therapeutic strategies for women’s health.

## 1. Introduction

Sedentary behavior and insufficient physical activity represent pervasive global health challenges. Approximately 27.5% of adults worldwide fail to meet recommended physical activity levels,^[1]^ while sedentary time (particularly leisure screen time [LST]) continues to rise dramatically, especially in high-income countries.^[2]^ Concurrently, psychological well-being concerns, including mood instability and reduced subjective well-being, contribute to a substantial burden of disease, with women reporting a higher prevalence of mood disorders and psychological distress compared to men.^[3–5]^ These interconnected movement behaviors and psychological states are increasingly recognized as potentially modifiable determinants of multiple chronic conditions.^[6,7]^ For women’s health specifically, extensive observational research suggests associations between these factors and a range of reproductive disorders. Higher levels of physical activity have been linked to a reduced risk of breast cancer,^[8,9]^ endometriosis,^[10]^ and potentially polycystic ovary syndrome (PCOS),^[11]^ while both sedentary behavior and psychological distress have been observationally associated with an increased risk of these conditions, as well as infertility and adverse pregnancy outcomes.^[2,6,12–15]^

However, the causal nature of these associations remains contentious. Observational studies are inherently limited by residual confounding and reverse causation.^[16]^ For instance, pain from endometriosis may restrict physical activity, or hormonal imbalances in PCOS could contribute to mood disturbances. While prior Mendelian randomization (MR) studies have begun to address these limitations by confirming a causal link for an individual factor like physical activity with some gynecologic conditions,^[17,18]^ the independent effects of movement behaviors and the distinct causal roles of psychological well-being remain largely unresolved and their systematic interplay across a comprehensive spectrum of obstetric and gynecologic diseases have not been established.^[19]^

Consequently, establishing the independent causal roles of these intertwined factors requires more robust methodological approaches. MR offers a powerful strategy to strengthen causal inference.^[16,20]^ Here, we leveraged a comprehensive framework combining univariable MR, multivariable Mendelian randomization (MVMR), and mediation analysis. This study aimed to systematically disentangle the independent effects of sedentary behavior, physical activity, mood instability, and well-being on a broad spectrum of 20 reproductive disorders and to explore the potential biological pathways mediating these associations. To our knowledge, this is the first study to comprehensively address these complex interrelationships in a single, integrated design, providing novel genetic evidence to inform targeted prevention and therapeutic strategies for women’s health.

While prior MR studies have established foundational causal evidence linking individual lifestyle factors, such as physical activity, to specific gynecologic outcomes including breast cancer and endometriosis,^[17,21]^ the etiological architecture of these associations remains incompletely understood. The existing literature is characterized by several critical limitations that the present study was designed to address. First, the preponderance of studies relies on univariable MR, an approach that cannot definitively delineate the independent contributions of interrelated exposures, such as sedentary behavior versus physical activity.^[21,22]^ Second, the potential causal role of psychological determinants, while investigated in isolation,^[23]^ has not been concurrently evaluated alongside movement behaviors within a unified analytical framework, precluding an assessment of their distinct versus shared etiological pathways. Finally, there is a marked paucity of research employing MR-based mediation analysis to systematically elucidate the biological mechanisms that may underlie these relationships.^[24,25]^

To address these lacunae, the present study leverages a comprehensive MVMR and mediation analysis framework. We aim to deconvolve the independent causal effects of sedentary behavior, physical activity, mood instability, and well-being across an extensive repertoire of 20 gynecologic and obstetric disorders, while simultaneously providing novel mechanistic insights to inform the etiology and primary prevention of major women’s health conditions.

## 2. Methods

**Figure 1.**
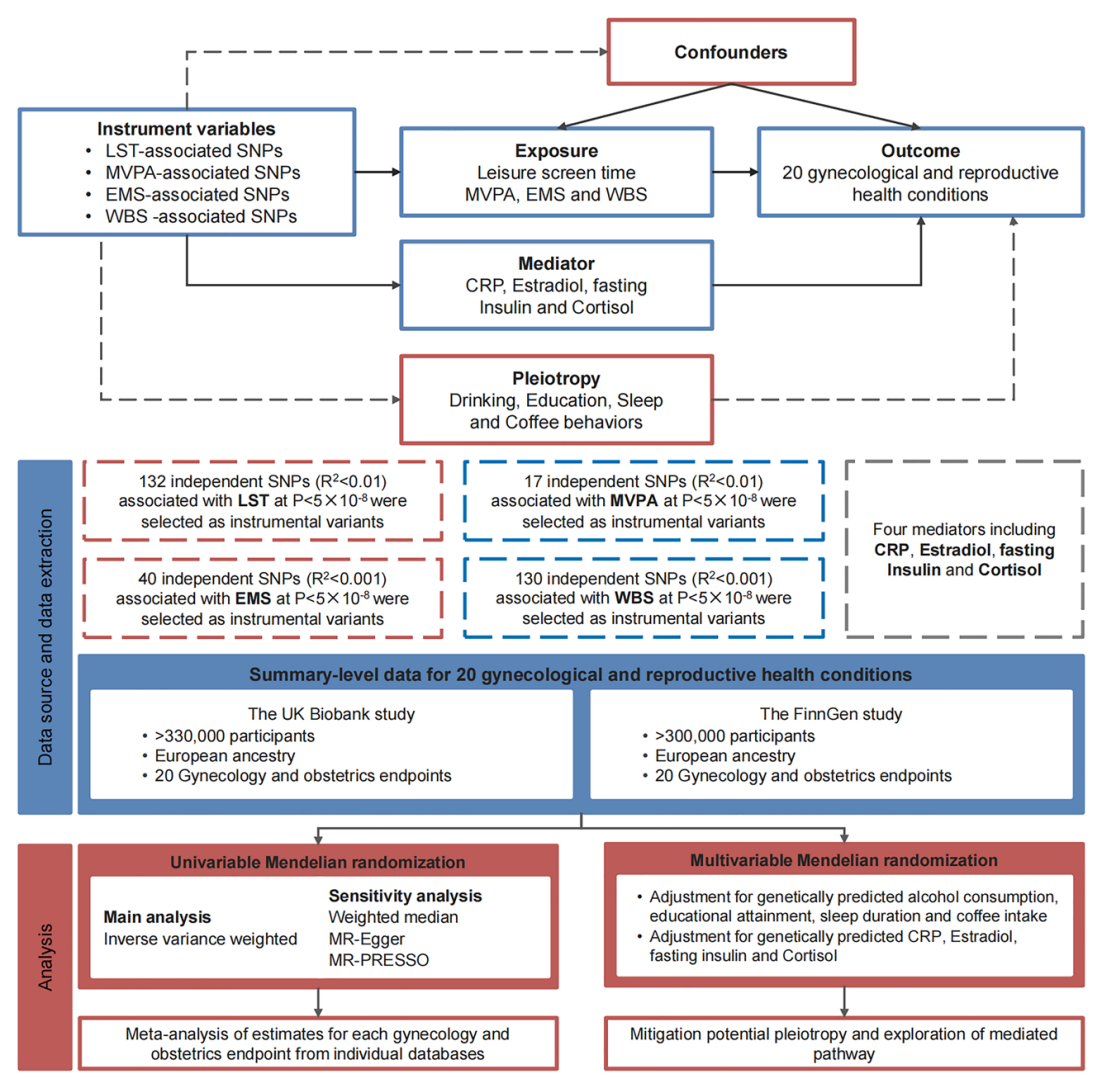
Study design. CRP = C-reactive protein levels, EMS = experiencing mood swings, LST = leisure screen time, MR = Mendelian randomization, MR PRESSO = Mendelian randomization pleiotropy residual sum, and outlier, MVPA = moderate-to-vigorous intensity physical activity during leisure time, SNP = single nucleotide polymorphism, WBS = well-being spectrum.

We defined LST as an indicator of a sedentary lifestyle and moderate-to-vigorous intensity physical activity (MVPA) as a measure of physical activity. Concurrently, we used experiencing mood swings (EMS) and the well-being spectrum (WBS) as proxies for psychological states. Figure 1 depicts the overall study design. This investigation utilized summary-level data from large genome-wide association studies (GWAS), primarily the UK Biobank (UKB) and FinnGen studies,^[26,27]^ with detailed data source information available in Table S1, Supplemental Digital Content. For each gynecologic and obstetric endpoint, We first generated MR estimates within individual outcome datasets and subsequently combined them via meta-analysis. In our primary analysis, we employed univariable MR to investigate the associations between the 4 genetically predicted exposures and the risks of 20 gynecologic and obstetric disorders. This approach relies on 3 core assumptions: the genetic variants used as instruments are robustly associated with the exposure; the genetic instruments are not associated with confounders; and the instruments influence the outcome exclusively through the exposure. To mitigate potential pleiotropy from confounders, specifically, alcohol consumption, coffee intake, educational attainment, and sleep duration, we applied MVMR to the identified associations. Finally, we examined the potential mediating effects of C-reactive protein (CRP), estradiol, fasting insulin, and cortisol on the exposure–outcome relationships.

### 2.1. Instrumental variable selection

We extracted genetic variants for LST (N = 606,820) and MVPA (N = 526,725) from a comprehensive GWAS meta-analysis of 51 European populations.^[28]^ The source GWAS for these 2 exposures accounted for family relatedness and adjusted for age, age-squared, principal components of population structure, and other study-specific covariates. Concurrently, we obtained genetic instruments for EMS (N = 373,733) from a large-scale GWAS on neuroticism traits^[29]^ and for the WBS (N = 2083,151) from a multivariate genome-wide association meta-analysis.^[30]^

For all 4 exposures, we selected single nucleotide polymorphisms (SNPs) that reached genome-wide significance (*P* < 5 × 10^−^8) as initial instrumental variables. To ensure independence, these SNPs were subjected to linkage disequilibrium (LD) clumping based on a threshold of *r*^2^ < 0.01 within a 10,000 kb window, a standard criterion chosen to balance the removal of correlated variants with the retention of sufficient instruments for statistical power. This procedure yielded 132, 17, 40, and 130 independent SNPs for LST, MVPA, EMS, and WBS, respectively. Table S2, Supplemental Digital Content provides detailed information on these selected SNPs. Furthermore, to test the robustness against potential residual LD, we conducted a sensitivity analysis using instruments selected under a more stringent LD threshold (*r*^2^ < 0.001). Finally, to mitigate pleiotropy, we utilized the PhenoScanner V2 database^[31]^ to identify and exclude any instrumental SNPs that were also strongly associated with the outcomes.

### 2.2. Gynecologic and obstetric disease data sources

Genetic association data for 20 gynecologic and obstetric disorders were primarily sourced from the UKB study and the FinnGen consortium (data release R12).^[31,32]^ The UKB, a large-scale multicenter prospective cohort that enrolled approximately 500,000 participants in the United Kingdom from 2006 to 2010, provided the primary outcome data for our study. For this cohort, we utilized summary-level GWAS statistics from the Lee Lab (https://www.leelabsg.org/resources) for European-ancestry individuals, wherein endpoints were defined by International Classification of Diseases (ICD-9 and ICD-10) codes. The analysis of these genetic associations accounted for sex, birth year, and the first 4 genetic principal components as covariates. Correspondingly, data from the FinnGen consortium were utilized, which integrates genetic information from nationwide biobanks with disease status from structured national health registers in Finland. In FinnGen, outcomes were defined via ICD-10 codes, and the genetic associations were adjusted for sex, age, genotyping batch, and 10 principal components. The specific ICD codes used to define all outcomes in both UKB and FinnGen are detailed in Table S3, Supplemental Digital Content.

### 2.3. Data sources for confounders and mediators

Genetic instruments for potential confounders and mediators were obtained from the latest publicly available GWAS summary statistics of European ancestry. These included: alcohol consumption (drinks per week; Saunders et al, N = 632,802),^[33]^ educational attainment (Okbay et al, N = 405,072),^[34]^ sleep duration (Jones et al, N = 127,573),^[35]^ coffee intake (Schoeler et al, N = 263,463),^[36]^ CRP levels (Koskeridis et al, N = 575,531),^[37]^ estradiol levels (Schmitz et al, N = 163,947),^[38]^ fasting insulin levels (Meta-analysis of Glucose and Insulin-related traits Consortium, N = 50,404),^[39]^ and cortisol levels (Cortisol Network Consortium, N = 25,314).^[40]^ Detailed information is provided in Table S1, Supplemental Digital Content.

### 2.4. Statistical analysis

Our analytical strategy began with estimating the total causal effect of each exposure on each outcome using the inverse-variance weighted (IVW) method under a multiplicative random-effects model. For outcomes with data from both the UKB and FinnGen, estimates were combined using a fixed-effects meta-analysis, unless significant heterogeneity was present (Cochran *Q P* < .05), in which case a random-effects model was employed. To assess the robustness of our findings and test for violations of MR assumptions, a suite of sensitivity analyses was conducted, including the weighted median method, MR-Egger regression, Mendelian Randomization Pleiotropy RESidual Sum and Outlier (MR-PRESSO), and leave-one-out analysis. The causal direction of the observed associations was verified using the MR-Steiger test.

To dissect the independent effects of correlated exposures, we performed 2-way MVMR with mutual adjustment for paired exposures: LST versus MVPA, and EMS versus the WBS. This approach allowed us to estimate the effect of one exposure while accounting for the genetic influence of the other. Furthermore, to assess the robustness of these estimates, a series of additional MVMR analyses were conducted. Specifically, each primary exposure was systematically and individually adjusted for each of the 4 genetically proxied potential confounders (alcohol consumption, educational attainment, sleep duration, and coffee intake), a strategy chosen to precisely examine the influence of each confounder while mitigating risks of multicollinearity.

The MVMR framework was also utilized for an exploratory mediation analysis to investigate potential underlying biological pathways. This was achieved by adjusting the exposure–outcome associations for genetically predicted levels of key mediators, including an inflammatory marker (CRP), hormonal factors (estradiol, cortisol), and a metabolic marker (fasting insulin). The proportion of the total effect mediated by a specific factor was estimated using the formula: 1 − direct effect (the estimate after adjusting for the mediator)/total effect (the estimate in the univariable MR analysis), where the direct effect is the estimate from the mediator-adjusted MVMR analysis, and the total effect is the estimate from the univariable MR analysis. To account for multiple testing, all mediation *P*-values were adjusted using the Benjamini–Hochberg procedure to control the false discovery rate (FDR). We acknowledge the statistical limitations of the proportion mediated formula and the assumption of no confounding of the mediator–outcome pathway; therefore, these findings are presented as exploratory and should be interpreted with caution.

Power calculations were performed for each primary analysis (Table S5, Supplemental Digital Content), a crucial step to ensure a rigorous assessment of our estimates. The strength of the genetic instruments was evaluated using the *F*-statistic, with a value >10 indicating a sufficiently strong instrument. Conditional *F*-statistics for the MVMR analyses were also calculated to assess instrument strength and are presented in Table S12, Supplemental Digital Content. The odds ratios (ORs) and 95% confidence intervals (CIs) were scaled to represent the effect per one standard deviation increase in each exposure. To address multiple testing, we applied the Benjamini–Hochberg procedure to control the FDR. Associations with an FDR-adjusted *P*-value (*q*-value) < 0.05 were considered statistically significant. All analyses were 2-sided and performed using R (version 4.3.2; R Foundation for Statistical Computing, Vienna, Austria) with the “TwoSampleMR,” “MendelianRandomization,” and “MRPRESSO” packages.

## 3. Results

**Figure 2.**
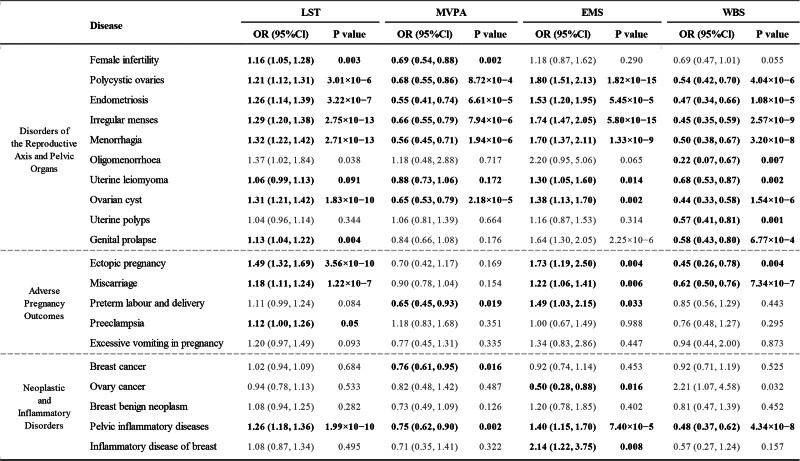
Complete results of univariable Mendelian randomization analyses for 4 exposures on 20 gynecologic and obstetric disorders. The table presents the primary causal estimates from the inverse-variance weighted meta-analysis. Results are presented as odds ratios (OR) with 95% confidence intervals (CI) and corresponding *P*-values per one standard deviation increase in each genetically predicted exposure. Associations that were not statistically significant after FDR correction (*q* ≥ 0.05) are indicated in gray font. Diseases are grouped by clinical category. EMS = experiencing mood swings, FDR = false discovery rate, LST = leisure screen time, MVPA = moderate-to-vigorous physical activity, WBS = well-being spectrum.

**Figure 3.**
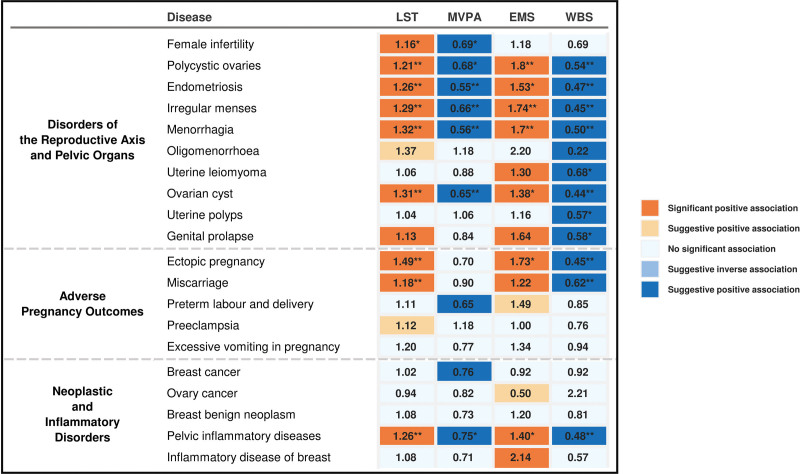
Heatmap of causal associations between 4 lifestyle and psychological factors and 20 gynecologic and obstetric disorders. The heatmap displays the odds ratios (ORs) from the primary inverse-variance weighted Mendelian randomization analyses. Each cell represents the causal effect of a specific exposure (columns) on a specific disorder (rows). The color scheme is designed to convey both the direction and the statistical significance of the associations. The color hue indicates the direction of the effect: orange tones represent risk-increasing associations (OR > 1), while blue tones represent risk-reducing associations (OR < 1). Crucially, the color saturation indicates the level of statistical significance: saturated (deep) colors are used for associations that remained statistically significant after FDR correction (*q* < 0.05), whereas lighter shades are used for associations that were significant only at a nominal threshold (*P* < .05) but did not survive FDR correction. Uncolored cells indicate no significant association (*P* ≥ .05). Asterisks within the cells denote the FDR-corrected significance level: *q < 0.05; **q < 0.001. EMS = experiencing mood swings, FDR = false discovery rate, LST = leisure screen time, MVPA = moderate-to-vigorous physical activity, WBS = well-being spectrum.

Genetically predicted LST and EMS were associated with an increased risk for a broad range of gynecologic and obstetric disorders, whereas MVPA and a higher WBS score were generally protective. A comprehensive summary of all primary univariable MR findings that remained significant after FDR correction, derived from a meta-analysis of estimates from the UKB and FinnGen cohorts (Fig. 2). These causal patterns are further visualized in the heatmap (Fig. 3).

All genetic instruments used for the exposures exhibited robust strength (*F*-statistics >10; Table S2, Supplemental Digital Content), and our study was well-powered to detect ORs generally ranging from 1.13 to 2.88 at 80% power (Table S5, Supplemental Digital Content). Potential pleiotropy was minimized through PhenoScanner analysis (Table S6, Supplemental Digital Content), and the robustness of the identified associations was supported by the MR-Steiger test, which confirmed the assumed causal direction (Table S4, Supplemental Digital Content). Detailed results for each exposure are described below.

### 3.1. Associations between LST and gynecologic and obstetric disorders

Genetically predicted LST was causally associated with an increased risk for 10 of the 20 investigated disorders (FDR *q* < 0.05; Table S7, Supplemental Digital Content). The strongest associations were observed for ectopic pregnancy (OR 1.49, 95% CI: 1.32–1.69; *P* = 3.56 × 10^−10^) and ovarian cyst (OR 1.31, 95% CI: 1.21–1.42; *P* = 1.83 × 10^−11^), with other notable risk-increasing effects on conditions such as endometriosis (OR 1.26, 95% CI: 1.14–1.39; *P* = 3.22 × 10^−6^) and polycystic ovaries (OR 1.21, 95% CI: 1.12–1.31; *P* = 3.01 × 10^−6^) (Fig. 2; Table S8, Supplemental Digital Content).

A series of sensitivity analyses were conducted to assess the robustness of these findings (Table S8, Supplemental Digital Content). Although evidence of between-instrument heterogeneity was observed within the FinnGen dataset for 9 outcomes (Cochran *Q P* < .05), the results from robust methods, including the Weighted Median and MR-Egger, remained largely consistent. The MR-Egger intercept analysis identified a signal for potential directional pleiotropy only for the association with genital prolapse (*P*-intercept = .017). For this specific outcome, the risk-increasing effect remained directionally consistent in the weighted median analysis (OR 1.22, 95% CI: 1.07–1.39). Furthermore, the MR-PRESSO analysis detected and removed outliers for 8 of the 10 associations, but all findings retained their statistical significance post-correction, including for genital prolapse. The MR-Steiger test confirmed the correct causal direction for all 10 associations (Table S4, Supplemental Digital Content).

In the pairwise MVMR analysis, after mutual adjustment for genetically predicted MVPA, the associations between LST and 9 of the 10 disorders remained statistically significant, with only slight attenuation of the ORs. The association with female infertility was attenuated and no longer remained significant after FDR correction in the MVMR model (FDR adjusted *P* = .054). The full results of the univariable and multivariable analyses for LST are visualized in Figure S3, Supplemental Digital Content.

### 3.2. Associations between MVPA and gynecologic and obstetric disorders

Genetically predicted MVPA was causally associated with a decreased risk for 9 of the 20 investigated disorders (FDR *q* < 0.05; Table S7, Supplemental Digital Content). The most pronounced protective effects were observed for endometriosis (OR 0.55, 95% CI: 0.41–0.74; *P* = 6.61 × 10^−5^) and menorrhagia (OR 0.56, 95% CI: 0.45–0.71; *P* = 1.94 × 10^−6^). MVPA also demonstrated significant protective associations with conditions including polycystic ovaries (OR 0.68, 95% CI: 0.55–0.86; *P* = 8.72 × 10^−4^), irregular menses (OR 0.66, 95% CI: 0.55–0.79; *P* = 7.94 × 10^−6^), and breast cancer (OR 0.76, 95% CI: 0.61–0.95; *P* = .016) (Fig. 2; Table S9, Supplemental Digital Content).

Sensitivity analyses were performed to probe the robustness of these 9 associations (Table S9, Supplemental Digital Content). For outcomes with evidence of between-instrument heterogeneity (polycystic ovaries, menorrhagia, and endometriosis), the protective effects remained directionally consistent and significant across both Weighted Median and MR-Egger analyses. A notable finding emerged for breast cancer, where the MR-Egger analysis suggested a potential risk-increasing effect (OR 1.54, 95% CI: 0.48–4.93), directionally opposite to the primary IVW estimate, although this was not statistically significant and the intercept test showed no evidence of pleiotropy (*P*-intercept = .170). Another inconsistency was observed for pelvic inflammatory diseases, which showed a signal for directional pleiotropy (*P*-intercept = .036), and for which the protective association did not remain significant in either the weighted median or MR-Egger modZoom-20251015 1153-2.docxels. For all other analyses, the MR-PRESSO global test for pleiotropy was nonsignificant, and the MR-Steiger test confirmed the correct causal direction. Furthermore, leave-one-out analysis confirmed that the identified causal associations for MVPA were not driven by any single genetic instrument (Figure S1, Supplemental Digital Content).

In the pairwise MVMR analysis mutually adjusting for LST, the causal estimates for MVPA were altered for several outcomes (Figs. 1 and 3 and Figure S3, Supplemental Digital Content). The protective associations for preterm labor and delivery and breast cancer were attenuated to null after accounting for LST. Conversely, after adjusting for the confounding effect of LST, MVPA emerged as a protective factor for 3 additional outcomes: genital prolapse (OR 0.80, 95% CI: 0.69–0.93), breast benign neoplasm (OR 0.59, 95% CI: 0.45–0.78), and uterine leiomyoma (OR 0.85, 95% CI: 0.75–0.96).

### 3.3. Associations between EMS and gynecologic and obstetric disorders

Genetically predicted EMS showed significant positive associations with 12 gynecological conditions in IVW meta-analysis: polycystic ovaries (OR 1.80, 95% CI: 1.51–2.13; *P* = 1.82 × 10^−11^), ovarian cyst (OR 1.38, 95% CI: 1.13–1.70; *P* = .002), pelvic inflammatory diseases (OR 1.40, 95% CI: 1.15–1.70; *P* = 7.40 × 10^−4^), genital prolapse (OR 1.64, 95% CI: 1.30–2.05; *P* = 2.25 × 10^−5^), miscarriage (OR 1.22, 95% CI: 1.06–1.41; *P* = .006), inflammatory breast disease (OR 2.14, 95% CI: 1.22–3.75; *P* = .008), ectopic pregnancy (OR 1.73, 95% CI: 1.19–2.50; *P* = .004), irregular menses (OR 1.74, 95% CI: 1.47–2.05; *P* = 5.80 × 10^−11^), menorrhagia (OR 1.70, 95% CI: 1.37–2.11; *P* = 1.33 × 10^−6^), endometriosis (OR 1.53, 95% CI: 1.20–1.95; *P* = 5.45 × 10^−4^), and uterine leiomyoma (OR 1.30, 95% CI: 1.05–1.60; *P* = .014) (Fig. 2; Table S7, Supplemental Digital Content).

The robustness of these associations was strongly supported by a comprehensive suite of sensitivity analyses (Table S10, Supplemental Digital Content). Although between-instrument heterogeneity was observed in the FinnGen dataset for genital prolapse and menorrhagia, the risk-increasing effects for these outcomes remained directionally consistent across all robust MR methods. Crucially, the MR-Egger intercept analysis revealed no evidence of directional horizontal pleiotropy for any of the 12 significant associations (all *P*-intercepts >.05). Furthermore, while the MR-PRESSO analysis identified 2 potential outliers for the association with menorrhagia, the global test for pleiotropy was not significant (*P* = .781), indicating that these outliers did not systematically bias the causal estimate. The MR-Steiger test consistently confirmed the correct causal direction for all associations.

In the pairwise MVMR analysis mutually adjusting for the genetically predicted WBS, all 12 significant associations for EMS remained robust, with only minor fluctuations in the effect sizes (Fig. 3; Figure S3, Supplemental Digital Content). The direction and magnitude of these associations were largely unchanged after accounting for WBS.

### 3.4. Associations between WBS and gynecologic and obstetric disorders

Genetically predicted WBS demonstrated widespread protective effects, with significant causal associations observed for a decreased risk of 12 of the 20 investigated disorders (FDR *q* < 0.05; Table S7, Supplemental Digital Content). The protective effects were particularly pronounced for endocrine and menstrual-related disorders, with the largest effect sizes seen for oligomenorrhoea (OR 0.22, 95% CI: 0.07–0.67; *P* = .007), ovarian cyst (OR 0.44, 95% CI: 0.33–0.58; *P* = 1.54 × 10^−8^), and irregular menses (OR 0.45, 95% CI: 0.35–0.59; *P* = 2.57 × 10^−9^) (Fig. 2; Table S11, Supplemental Digital Content).

Sensitivity analyses revealed a complex picture regarding the robustness of these associations (Table S11, Supplemental Digital Content). Significant between-instrument heterogeneity was observed in the meta-analyses of 8 associations. For pelvic inflammatory diseases, the MR-Egger analysis suggested a signal for potential pleiotropy (*P*-intercept = .023) and indicated a directionally inconsistent, nonsignificant effect (OR 2.95, 95% CI: 0.56–15.42; *P* = .202). However, this association is provisionally retained because the MR-PRESSO global test for pleiotropy was highly significant (*P* = 5.50 × 10^−8^), and the outlier-corrected estimate remained significant and directionally consistent with the primary IVW analysis (OR 0.41, 95% CI: 0.31–0.54). For the other outcomes, MR-PRESSO analyses confirmed that the presence of outliers did not alter the overall findings post-correction. The MR-Steiger test supported the correct causal direction for all 12 primary associations.

In the pairwise MVMR analysis mutually adjusting for EMS, all 12 protective associations for WBS remained robust (Fig. 3; Figure S3, Supplemental Digital Content). Notably, after adjusting for the risk-increasing effect of EMS, a protective association emerged for female infertility (OR 0.66, 95% CI: 0.46–0.93), which was not significant in the univariable analysis.

### 3.5. Robustness of associations in multivariable analyses

The MVMR analyses revealed a high degree of robustness for many primary associations across a comprehensive suite of adjustment models (Figure S2, Supplemental Digital Content). Notably, a core set of 6 gynecologic disorders consistently emerged as being associated with all 4 exposures. For polycystic ovaries, ovarian cyst, pelvic inflammatory diseases, irregular menses, menorrhagia, and endometriosis, the risk-increasing effects of LST and EMS, alongside the protective effects of MVPA and WBS, were largely maintained irrespective of adjustment.

For LST specifically, its detrimental effects remained significant for nearly all 10 of its primary associations, with 7 outcomes (including ovarian cyst and ectopic pregnancy) demonstrating consistent significance across all 12 adjustment models (Figure S2, Supplemental Digital Content). For MVPA, its protective effects were also highly robust, particularly for irregular menses, menorrhagia, and endometriosis, which remained significant against all adjustments. The psychological exposures demonstrated even greater stability. The risk-increasing effects of EMS and the protective effects of WBS remained largely robust across all adjustments for their respective 12 significant associations. This stability was particularly evident for the 10 shared outcomes (including genital prolapse and uterine leiomyoma), where they exhibited mirrored, directionally opposite effects even after mutual adjustment. The detailed results of the adjustments for key lifestyle confounders are provided in Table S13, Supplemental Digital Content.

### 3.6. Mediation analyses

**Figure 4.**
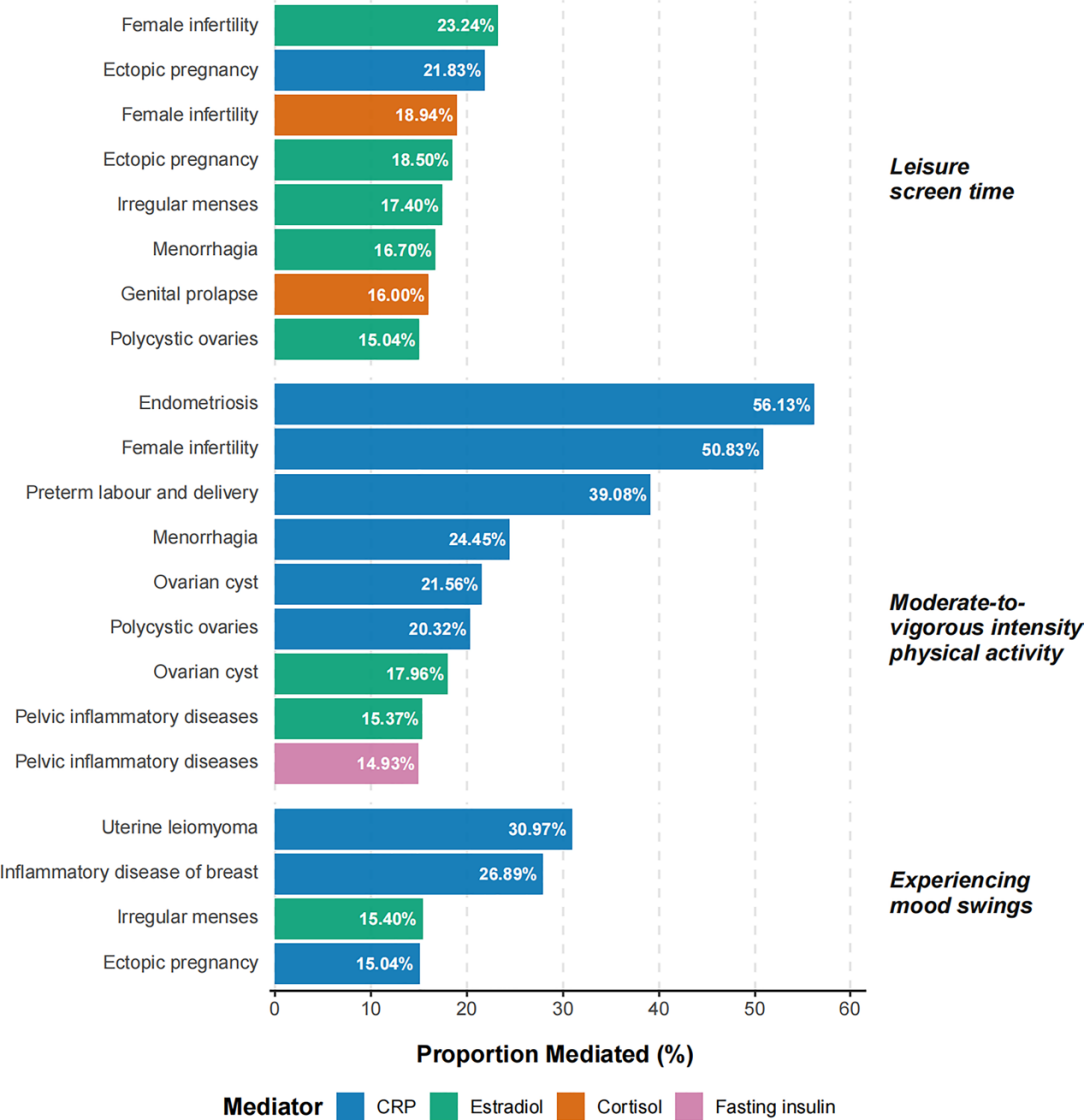
Exploratory analysis of key mediating pathways for the causal effects of lifestyle and psychological factors. This bar chart visually summarizes the most substantial mediation pathways identified in our study. Each bar represents the point estimate of the proportion (%) of a total causal effect that is mediated by a specific biological marker. Pathways are grouped by the primary exposure (leisure screen time, moderate-to-vigorous physical activity, and experiencing mood swings) and are ordered within each group by the magnitude of the mediation proportion. The color of each bar corresponds to the specific mediator. To focus on the most robust and noteworthy findings, only pathways that met 2 stringent criteria are presented: a statistically significant mediation effect after FDR correction (*q* < 0.05), and a substantial estimated mediation proportion (generally >15%). It is critical to note that these analyses are exploratory. The proportion mediated is an approximation, and these findings rely on the untestable assumption of no confounding of the mediator–outcome pathway. Therefore, these point estimates should be interpreted with significant caution as hypothesis-generating, and formal confidence intervals are not presented. The full, detailed results of all mediation analyses are available in Table S14, Supplemental Digital Content. CRP = C-reactive protein, FDR = false discovery rate.

To explore potential biological mechanisms underlying the primary associations, we conducted a systematic, exploratory mediation analysis. We identified numerous significant mediating pathways that remained robust after FDR correction (all *q* < 0.05). The most substantial of these findings, defined as pathways with a mediation proportion generally >15%, are visually summarized in Figure 4. The full results of all mediation tests are available in Figure S4, Supplemental Digital Content.

For LST, the top 3 mediating pathways were observed for its association with female infertility (mediated by estradiol; proportion ≈ 23.24%, *q* = 0.030), ectopic pregnancy (CRP; 21.83%, *q* = 2.34 × 10^−6^), and again with female infertility (cortisol; 18.94%, *q* = 0.033).

For MVPA, CRP emerged as the predominant mediator. The 3 strongest pathways identified were for the protective effects on endometriosis (56.13%, *q* = 1.15 × 10^−6^), female infertility (50.83%, *q* = 0.001), and preterm labor and delivery (39.08%, *q* = 0.027), all mediated by CRP.

For EMS, the most prominent mediation was observed for the effect on uterine leiomyoma, with an estimated 30.97% of the association mediated by CRP (*q* = 0.020). This was followed by the CRP-mediated pathway for inflammatory disease of the breast (26.89%, *q* = 0.016) and the estradiol-mediated pathway for irregular menses (15.40%, *q* = 3.39 × 10^−5^).

For the WBS, the identified mediation proportions that remained significant after FDR correction were generally modest. The top 3 significant mediating pathways were observed for the protective effects on miscarriage (estradiol; 12.72%, *q* = 9.59 × 10^−4^), ectopic pregnancy (CRP; 12.72%, *q* = 0.011), and polycystic ovaries (estradiol; 10.36%, *q* = 8.22 × 10^−4^). Although a higher point estimate for the mediation proportion was observed for uterine polyps (35.43%), this pathway did not survive FDR correction (*q* = 0.056).

## 4. Discussion

This MR study provides genetically strengthened evidence for causal relationships between sedentary behavior, physical activity, psychological well-being, and a comprehensive array of female reproductive and obstetric disorders, transcending the limitations of observational epidemiology.^[16,20]^ By investigating 20 diverse outcomes, including endometriosis, polycystic ovaries, and menstrual irregularities, we extend prior associations to a broader gynecological spectrum, addressing a critical gap in causal inference.^[11,41]^ MVMR analyses reveal independent effects of LST, MVPA, EMS, and WBS, providing nuanced insights into their distinct contributions to disease etiology.

Prolonged LST causally elevates risks across multiple reproductive outcomes, aligning with extensive epidemiological evidence linking sedentary behavior to metabolic and gynecological disorders.^[1,2]^ Conversely, MVPA mitigates susceptibility, notably for endometriosis and breast cancer, consistent with observational and MR studies.^[8,42]^ Our findings related to breast cancer may serve as an informative example of the utility of the MVMR framework. While univariable MR indicated a protective effect of MVPA on breast cancer risk, consistent with prior reports,^[8,42]^ this association was attenuated after adjusting for LST in the MVMR analysis. This observation does not discount the benefits of physical activity but rather suggests a more intricate relationship, where part of the protective effect nominally attributed to MVPA could potentially be explained by a concomitant reduction in sedentary time. Separating the independent effects of these behaviors is a known challenge in observational research due to their high correlation.^[43]^ By applying MVMR, our study attempts to address this issue, providing genetic evidence that may support the consideration of both increasing physical activity and reducing sedentary time as separate components in breast cancer prevention strategies. This underscores how such analytical approaches can help inform more nuanced public health recommendations. These independent effects, robust in MVMR, underscore the necessity of interventions targeting both reduced sedentary time and increased physical activity, as endorsed by global health guidelines.^[44]^ Psychological factors are equally critical: EMS heightens risks for conditions like menstrual disorders, while WBS confers protection, supporting the salutogenic role of positive psychological states.^[7,13]^ The persistence of these associations after mutual adjustment highlights distinct mechanistic pathways, advocating for integrated mental health strategies in gynecological care.

Mediation analyses elucidate biological pathways linking lifestyle and psychological factors to reproductive health. Chronic inflammation, proxied by CRP, is a pivotal mechanism, particularly in endometriosis and breast cancer, where inflammatory cascades disrupt tissue homeostasis and promote pathogenesis.^[45,46]^ Sedentary behavior likely exacerbates low-grade inflammation, amplifying disease risk through cytokine-mediated pathways.^[47]^ Estradiol-driven dysregulation of the hypothalamic–pituitary–gonadal axis contributes to outcomes like ovarian cysts and genital prolapse, reflecting altered sex hormone signaling that disrupts ovarian and uterine function.^[48]^ Insulin signaling, disrupted in PCOS, mediates associations for MVPA and EMS, with aberrant pathways driving hyperandrogenism and ovulatory dysfunction.^[49]^ Cortisol, via the hypothalamic–pituitary–adrenal axis, links psychological stress to adverse outcomes such as miscarriage, highlighting stress-induced endocrine perturbations.^[50]^ These pathways may help to inform the prioritization of potential therapeutic targets for future investigation. For instance, they raise hypotheses that anti-inflammatory agents for endometriosis, hormonal therapies for PCOS, and stress-reduction interventions for pregnancy outcomes could be promising avenues for clinical research, thereby highlighting areas with potential for translational development.

The public health implications are substantial. Integrated interventions reducing LST, promoting MVPA, and enhancing psychological well-being could significantly alleviate the global burden of gynecologic and obstetric disorders, aligning with World Health Organization recommendations.^[44,51]^ Clinically, our findings suggest that targeting inflammatory and hormonal pathways could be a relevant strategy in the development of precision medicine for conditions like endometriosis or PCOS, while economically, lifestyle interventions could mitigate healthcare costs in high-income settings where sedentary behavior is prevalent.^[2]^ These findings support policy-driven initiatives, such as workplace wellness programs, to promote women’s reproductive health.

Methodologically, this study advances MR applications through MVMR, robust MR-Egger, MR-PRESSO, weighted median sensitivity analyses, and mediation analyses, leveraging UKB and FinnGen large-scale cohorts with strong genetic instruments. Restriction to European ancestry minimizes population stratification, enhancing internal validity.

However, several limitations warrant consideration. Horizontal pleiotropy, a potential violation of MR assumptions, may introduce bias, though MR-Egger, MR-PRESSO sensitivity analyses suggest minimal impact.^[52]^ The limited number of instrumental variables for MVPA (17 SNPs) may reduce precision, potentially leading to an underestimation of effect sizes, particularly for outcomes with modest associations.^[53]^ Consequently, our study may have had limited statistical power for these analyses, and any null findings for MVPA should be interpreted with particular caution. Sample overlap between exposure and outcome datasets (e.g., UKB) could bias estimates toward observational associations, though strong instruments (*F*-statistics >10) mitigate this risk.^[16]^ Self-reported measures of LST and MVPA are susceptible to recall and social desirability biases, potentially affecting exposure accuracy.^[1]^ The restriction to European ancestry limits generalizability to diverse populations, necessitating validation in non-European cohorts to ensure global relevance.^[54]^ Smaller sample sizes for certain outcomes may constrain statistical power, particularly for detecting modest effects. Finally, unmeasured mediators or confounders not captured in MVMR could influence findings, though genetic instruments reduce residual confounding compared to observational designs.

Future investigations should validate these findings in African and Asian cohorts to enhance global applicability and conduct randomized controlled trials to assess interventions combining reduced screen time, increased physical activity, and psychological well-being, targeting outcomes such as endometriosis and PCOS.^[16,44]^ Multi-omics approaches, integrating molecular profiling of inflammatory and hormonal pathways, could further clarify mechanisms, advancing precision medicine for reproductive health.^[52,55]^ Such research will underpin scalable interventions, including digital health platforms to curb sedentary behavior and community-based mental health programs, aligned with World Health Organization guidelines.^[44,51]^ In summary, this study pioneers causal inference across a comprehensive spectrum of gynecological outcomes using MVMR, elucidating potentially targetable pathways involving inflammation and hormonal dysregulation that may contribute to the risk of endometriosis and PCOS. By providing a rationale for digital health interventions and global health policies, it lays a strong foundation for alleviating the burden of female reproductive disorders.

## 5. Conclusion

In conclusion, this MR study provides genetically strengthened evidence for independent causal roles of movement behaviors and psychological states in the etiology of a wide spectrum of gynecologic and obstetric disorders. Our findings demonstrate that genetically proxied LST and mood instability are risk factors, while physical activity and well-being are protective factors for numerous conditions. Methodologically, our systematic application of pairwise MVMR was instrumental not only in confirming the independent effects of these interrelated factors but also in revealing their complex interplay, suggesting that reducing sedentary time and promoting physical activity may represent separate public health targets. Mechanistically, our exploratory mediation analysis offers novel, albeit preliminary, genetic support for the involvement of specific biological pathways, highlighting inflammation (via CRP) and hormonal dysregulation as key potential mediators. Collectively, these results underscore the necessity of an integrated approach that separately targets sedentary behavior, physical activity, and mental well-being to advance women’s health, while also providing avenues for future research into the identified mechanistic pathways.

## Acknowledgments

We thank the UK Biobank, the FinnGen study, the Ovarian Cancer Association Consortium, the Breast Cancer Association Consortium, the International Endocrine Consortium, and the CoLab Consortium for sharing data.

## Author contributions

**Conceptualization:** Yide Hu, Bo Wang.

**Data curation:** Yunbo Cao.

**Formal analysis:** Yide Hu, Yunbo Cao.

**Funding acquisition:** Bingdong Zhan.

**Investigation:** Yunbo Cao, Weiting Xia.

**Methodology:** Yunbo Cao, Junxu Li.

**Resources:** Junxu Li, Weiting Xia.

**Software:** Yide Hu, Hong Wu.

**Supervision:** Hong Wu.

**Validation:** Hong Wu, Bo Wang, Junxu Li, Weiting Xia, Bingdong Zhan.

**Visualization:** Bo Wang, Bingdong Zhan.

**Writing – review & editing:** Bingdong Zhan.




































